# Spectrophotometric Method for Simultaneous Estimation of Escitalopram Oxalate and Clonazepam in Tablet Dosage Form

**DOI:** 10.4103/0250-474X.59559

**Published:** 2009

**Authors:** R. B. Kakde, D. D. Satone

**Affiliations:** Department of Pharmaceutical Sciences, Rashtrasant Tukadoji Maharaj Nagpur University, Nagpur-440 033, India

**Keywords:** Clonazepam, escitalopram, simultaneous estimation, UV spectrophotometry

## Abstract

A simple, accurate and precise spectrophotometric method has been developed for simultaneous estimation of escitalopram oxalate and clonazepam in combined dosage form. Simultaneous equation method is employed for simultaneous determination of escitalopram oxalate and clonazepam from combined dosage forms. In this method, the absorbance was measured at 238 nm for escitalopram oxalate and 273 nm for clonazepam. Linearity was observed in range of 5-100 μg/ml and 5-50 μg/ml for escitalopram and clonazepam respectively. Recovery studies confirmed the accuracy of proposed method and results were validated as per ICH guidelines. The method can be used for routine quality control of pharmaceutical formulation containing escitalopram and clonazepam.

A new fixed dose combination containing escitalopram oxalate (ESC) and clonazepam (CLO) is available in market in tablet dosage form. ESC[1] is an orally administered selective serotonin reuptake inhibitor. It is the pure *S* enantiomer of racemic bicyclic phthalane derivative of citalopram. Chemically it is S-(+)-1-[3-(dimethylamino)propyl]-1-(p-fluorophenyl)-5 phthalancarbonitrile oxalate. CLO is a benzodiazepine derivative similar to diazepam, with marked antiepileptic properties[2]. It is official in USP[3] and BP[4]. Chemically it is 5-(2-chlorophenyl)-1, 3-dihydro-7-nitro-2H-1,4-benzodiazepine-2-one. Nexito Plus is the commercial product that has been developed and marketed to treat the depression associated with anxiety.

Literature reports many analytical methods for quantitative determination of ESC and CLO individually in fixed dosage forms. These methods include estimation of ESC by LC-MS[5] and CLO by UV[6], HPLC[7–14] and gas chromatography[15]. However, no method is yet reported for simultaneous estimation of ESC oxalate and CLO in pharmaceutical preparation. Hence, the spectroscopic methods have been developed to estimate these two drugs from tablet dosage form.

The instrument used for the present study was UV/Vis double beam spectrophotometer, (Model Shimadzu UV 2401PC) with 1 cm matched pair quartz cells. ESC oxalate and CLO were kindly supplied as a gift sample by Wockhardt Ltd., Aurangabad, India and NPIL, Pithampur, MP, India. All chemicals and reagents used were of AR grade and were purchased from Merck Chemicals, India.

Standard stock solution (1000 μg/ml) of ESC was prepared by dissolving 50 mg of ESC in 50 ml of methanol. Five milliliter of this stock solution was further diluted to 100 ml by 0.1N HCl to get final concentration of 50 μg/ml. CLO standard stock solution (100 μg/ml) was prepared by dissolving 5 mg of CLO in 50 ml of methanol. Five milliliter of this stock solution was further diluted to 100 ml by 0.1N HCl to get final concentration of 5 μg/ml. These diluted solutions were then scanned in the wavelength range of 200-400 nm (fig. 1). The overlain spectra of ESC and CLO showed the λ_max_ at 238 nm and 273 nm, respectively. Hence the two wavelengths were selected for estimation of ESC and CLO. For constructing calibration curves, two series of different concentration in the range of 1-200 μg/ml for ESC and 1-100 μg/ml for CLO were prepared in 0.1N HCl from stock solution. ESC and CLO obeyed linearity in the concentration range of 5-100 μg/ml and 5-50 μg/ml with correlation coefficient of 0.9996 and 0.9992, respectively. The absorptivities (E 1%, 1 cm) of both the drugs at two-selected wavelength were then determined. These calculated values were the mean of five independent determinations. Five binary mixture solutions of ESC and CLO (clinical dose ratio 5:0.5 mg of ESC and CLO) and the dilution of 5: 0.5 μg/ml were prepared. The quantitative estimation of these drugs were carried out by solving the simultaneous equations, C_X_ = (A_2_ay_1_-A_1_ay_2_)/(ax_2_ay_1_-ax_1_ay_2_) and Cy=(A_1_ax_2_-A_2_ax_1_)/(ax_2_ay_1_-ax_1_ay_2_), where, A_1_ and A_2_ are absorbance of diluted mixture at 238 and 273 nm, respectively, Cx and Cy are the concentration of ESC and CLO, respectively (g/100 ml), ax_1_ (506.37) and ax_2_ (58.98) are absorptivities of ESC at 238 and 273 nm, respectively, ay_1_ (323.47) and ay_2_ (621.47) are absorptivities of CLO at 238 and 273 nm, respectively.

**Fig. 1:**
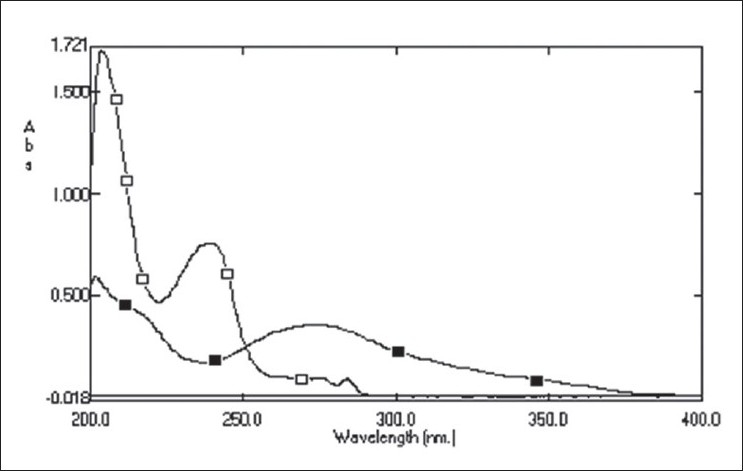
Overlain spectra of ESC and CLO Overlain spectra of escitalopram oxalate ESC (–□–) and clonazepam CLO (–■–) in 0.1N HCl

For analysis of marketed formulations, twenty tablets were accurately weighed; average weight was calculated and finally powdered. An amount equivalent to one tablet (5 mg of ESC and 0.5 mg of CLO) was taken and transferred to 50.0 ml volumetric flask and sonicated with 5 ml methanol. About 10 ml of 0.1N HCl was added and shaken for 30 min. The volume was made up to the mark with 0.1N HCl and the solution was mixed and filtered through Whatman grade I filter paper. The final solution containing 50 μg/ml of ESC and 5 μg/ml of CLO was used as sample solution. The above solution was then analyzed for the content of ESC and CLO using the method described above. The results of the marketed formulation are given in the Table 1.

**TABLE 1:** RESULTS OF MARKETED FORMULATION, RECOVERY AND INTERMEDIATE PRECISION

Drugs	Parameters	% Labeled claim**	% Recovery*	Intermediate Precision**		
				
				Interday	Intraday	Different analyst
ESC	Mean	99.07	100.47	100.79	99.75	98.92
	±SD	0.619	0.233	0.940	1.252	0.269
	% RSD	0.624	0.231	0.932	1.255	0.271
CLO	Mean	98.56	99.53	99.89	99.54	98.52
	±SD	0.597	0.416	0.470	0.710	0.264
	% RSD	0.605	0.418	0.470	0.713	0.267

* Mean of nine determination

** Mean of five determination, SD is the standard deviation, RSD is the relative standard deviation

Degradation study of sample solutions were carried out by exposing an accurately weighed quantity of tablet powder equivalent to about 50 mg of ESC oxalate and 0.5 mg of CLO to various stress conditions for 24 h like at 50° after addition of 1.0 ml of 0.1N HCl, at 50° after addition of 1.0 ml of 0.1N NaOH, at 50° after addition of 1.0 ml of 3.0 % H_2_O_2,_ at 60° and in a UV-cabinet at 265 nm. The degradation study showed that CLO was completely degraded in basic as well as acidic condition. The specificity data are summarized in Table 2.

**TABLE 2:** RESULTS OF SPECIFICITY DATA

Drug	Normal	Acid	Alkali	H_2_O_2_	UV	Heat
ESC	99.28	99.39	102.55	98.92	100.52	96.52
CLO	98.30	27.01	21.90	99.53	95.99	98.90

Determination of the limit of detection (LOD) and limit of quantitation (LOQ) is based on standard deviation of response and slope of calibration curve. LOD and LOQ of the methods were established according to ICH definitions- DL= 3.3σ/S, QL= 10σ/S, where DL- detection limit, QL- quantitation limit, σ-standard deviation of regression line, S-slope of the calibration curve. LOD at 238 nm was found to be 0.345, 1.01 and at 273 nm was 4.296, 1.09 for ESC and CLO, respectively. LOQ at 238 nm was found to be 1.045, 3.08 and at 273 nm was 13.01, 3.30 for ESC and CLO, respectively. System precision was determined by five replicate applications and five times measurement of laboratory mixture at the analytical concentration. This was performed by preparing laboratory mixtures of ESC and CLO in the ratio mentioned in marketed formulations. The results of the system precision were found to be 101.65±0.389 and 99.18±0.656 for ESC and CLO, respectively. Method precision was determined by analyzing the marketed formulations. The results of method precision were found to be 99.07±0.619 and 98.56±0.597 for ESC and CLO, respectively. The % RSD for inter-day and intra-day precision was found to be 0.932, 1.255 for ESC and 0.470, 0.713 for CLO, respectively. The results of recovery studies performed by standard addition method being close to 100% are indicative of the accuracy of the method and shows that the method is free from interference of excipients present in the formulation. The results of recovery study are given in Table 1. Ruggedness of the method was observed by reproducibility of results under the conditions like different days and different analyst. The method was found to be specific, accurate and precise. Hence the method can be employed for routine quality control of pharmaceutical formulation containing ESC and CLO.
